# Adult neurogenesis and “immature” neurons in mammals: an evolutionary trade-off in plasticity?

**DOI:** 10.1007/s00429-023-02717-9

**Published:** 2023-10-13

**Authors:** Luca Bonfanti, Chiara La Rosa, Marco Ghibaudi, Chet C. Sherwood

**Affiliations:** 1 Neuroscience Institute Cavalieri Ottolenghi, Orbassano, Italy; 2 Department of Veterinary Sciences, University of Turin, Largo Braccini 2, 10095 Turin, Grugliasco Italy; 3 Department of Anthropology and Center for the Advanced Study of Human Paleobiology, The George Washington University, Washington, DC USA

**Keywords:** Brain plasticity, Evolution, Mammals, Cerebral cortex, Neocortex, Olfactory bulb

## Abstract

Neuronal plasticity can vary remarkably in its form and degree across animal species. Adult neurogenesis, namely the capacity to produce new neurons from neural stem cells through adulthood, appears widespread in non-mammalian vertebrates, whereas it is reduced in mammals. A growing body of comparative studies also report variation in the occurrence and activity of neural stem cell niches between mammals, with a general trend of reduction from small-brained to large-brained species. Conversely, recent studies have shown that large-brained mammals host large amounts of neurons expressing typical markers of neurogenesis in the absence of cell division. In layer II of the cerebral cortex, populations of prenatally generated, non-dividing neurons continue to express molecules indicative of immaturity throughout life (cortical immature neurons; cINs). After remaining in a dormant state for a very long time, these cINs retain the potential of differentiating into mature neurons that integrate within the preexisting neural circuits. They are restricted to the paleocortex in small-brained rodents, while extending into the widely expanded neocortex of highly gyrencephalic, large-brained species. The current hypothesis is that these populations of non-newly generated “immature” neurons might represent a reservoir of developmentally plastic cells for mammalian species that are characterized by reduced stem cell-driven adult neurogenesis. This indicates that there may be a trade-off between various forms of plasticity that coexist during brain evolution. This balance may be necessary to maintain a “reservoir of plasticity” in brain regions that have distinct roles in species-specific socioecological adaptations, such as the neocortex and olfactory structures.

## Introduction

**Fig. 1:**
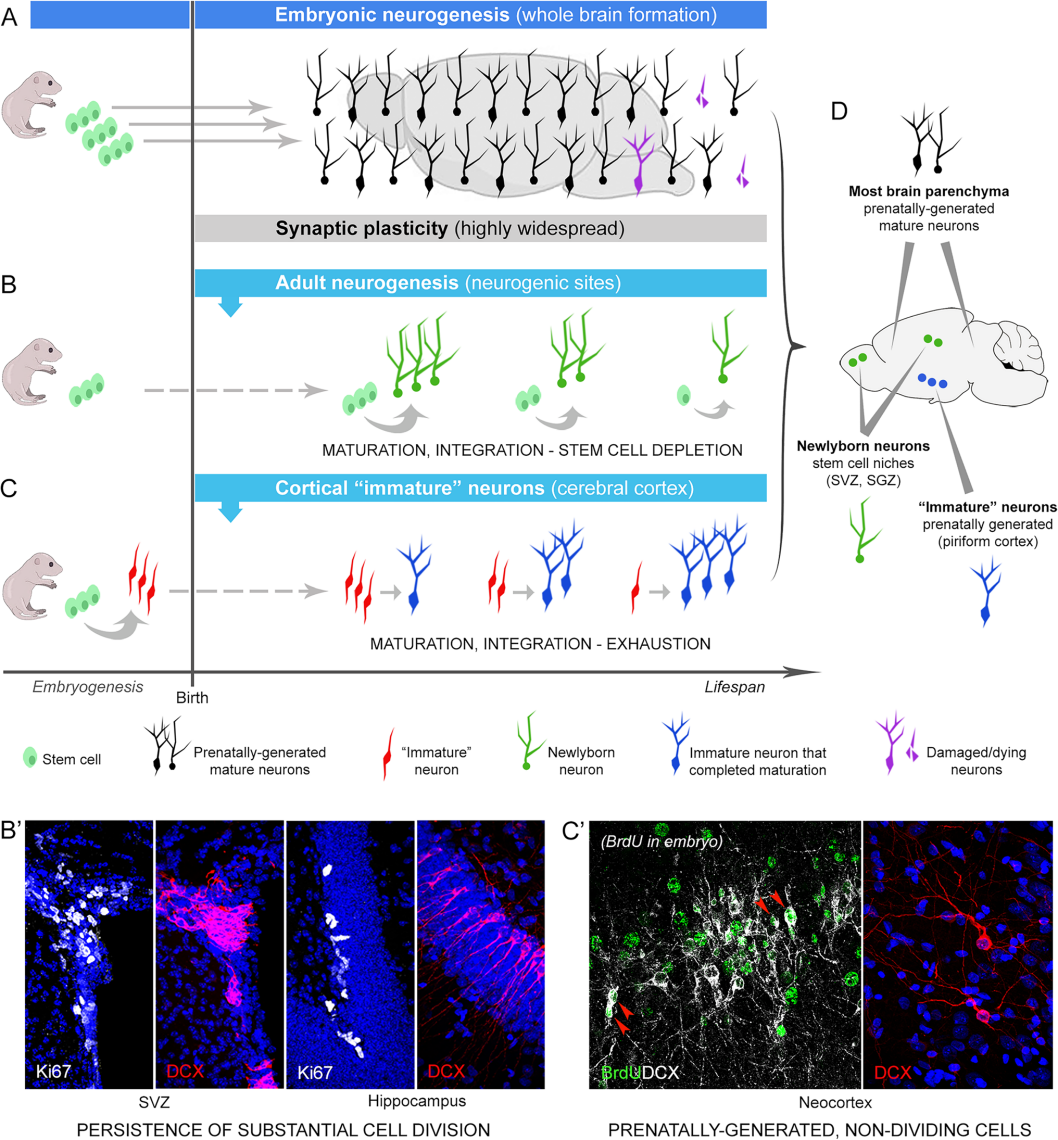
Neurogenesis as a process to build up the brain (dark blue), and to provide new neurons during adulthood at specific locations (light blue). **A**, the vast majority of brain neurons are produced during embryogenesis, then reach maturation during postnatal assembly and stabilization of the neural circuits (black). The life of these neurons spans the entire life of the animal, some of them undergoing damage/death because of aging or neurological diseases (purple). It is assumed that all these neurons can undergo synaptic plasticity (grey). **B**, neurogenic processes can last during adulthood in restricted neurogenic sites hosting stem cell niches (examples in B’). Full integration of functional mature neurons has been well documented in two brain sites: the olfactory bulb (from cells generated in the forebrain subventricular zone, SVZ) and the hippocampus (from cells generated in the subgranular zone of the dentate gyrus, SGZ). These processes undergo remarkable reduction through ages, due to stem cell depletion. **C**, neuronal integration of new elements in the circuits can also occur in the layer II of the cerebral cortex (piriform cortex in mice) through “awakening” and maturation of prenatally generated, “immature” neurons that had been blocked in an immature state since embryogenesis (examples in C’: left, DCX^+^ neurons in the rabbit neocortex; right, neocortical DCX^+^ neurons in lambs, generated during embryogenesis in pregnant sheep treated with the thymidine analogue bromodeoxyuridine, BrdU). This “neurogenesis without division” can occur in the absence of active stem cells, undergoing exhaustion only after maturation of the entire reserve. D, at least three types of mature neurons are present in the adult brain on the basis of their origin: most of them were generated during embryogenesis and reach maturity in early postnatal periods (black), others are generated from stem cells in the neurogenic sites (green), and others come from delayed maturation of “immature” neurons (blue). Confocal images reproduced with permission from Ghibaudi et al. 2023a (B’ and C’, left) and Piumatti et al. 2018 (C’, right)

**Fig. 2:**
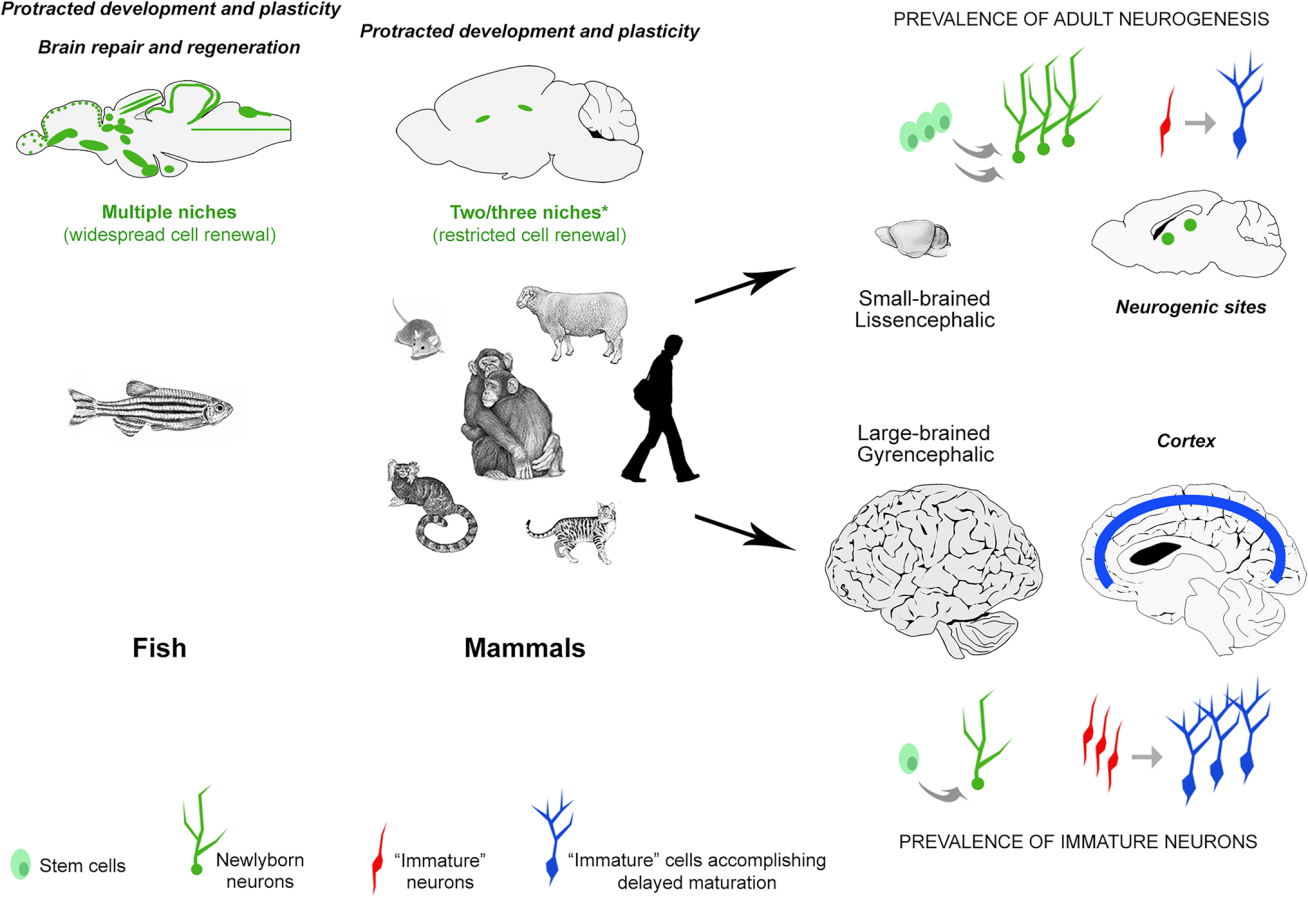
Heterogeneity, reduction, and specialization of brain structural plasticity in vertebrates. Top left, the amount, extension and activity of brain stem cell niches (green) vary remarkably among animals, being reduced from fish to mammals (asterisk: a third stem cell niche is described in the hypothalamus, the final fate/integration of newborn neurons being less studied). Right, the rate of neurogenesis, as well as its persistence through age, show variation among mammals, their reduction being more evident in large-brained compared with small-brained species. This trend is paralleled by a higher presence of non-newly generated, immature neurons (red and blue) in the cerebral cortex of large-brained species, suggesting a specialization (trade-off) of different types of plasticity in mammals

Neuronal plasticity is recognized as a crucial mechanism through which the central nervous system (CNS) learns from experience, forms memories, modifies the structure of neural networks over time, recovers after lesion or disease, and in some cases, regenerates lost nerve cells (Martino et al. 2011; Aimone et al. 2014; Bao and Song 2018; Obernier and Alvarez-Buylla 2019; Kempermann 2019; Bonfanti and Charvet 2021; La Rosa and Bonfanti 2021). Structural changes can impact the anatomy of the nervous system, from a subcellular to a neural circuit level. The most common type of structural remodeling is synaptic plasticity. It enables changes in connections between neurons, allowing the establishment of neural circuitry during development and subsequent refinement based on experience (Citri and Malenka 2008; Holtmaat and Svoboda 2009; Fig. 1A). This form of plasticity is expected to take place in nearly all parts of the grey matter in the central nervous system (CNS) and is likely well-conserved among mammals, reflected, in part, by the low interspecies variation of synaptic density and structure (apart from some differences probably linked to evolutionary adaptations of neural circuits to particular functions; Sherwood et al. 2020; De Felipe et al. 2002; Alonso-Nanclares et al. 2022). The most striking form of plasticity is adult neurogenesis, namely the formation of new neurons in specific neurogenic regions, as the result of neural stem cell activity (Aimone et al. 2014; Lim and Alvarez-Buylla 2016; Bao and Song 2018; Obernier and Alvarez-Buylla 2019; Fig. 1B). Since its discovery in mammals (Altman and Das 1965; Lois and Alvarez-Buylla 1994), adult neurogenesis has raised considerable interest, and it has been intensively studied with the objective of fostering therapeutic interventions aimed at brain repair, possibly harnessing the regenerative potential of neural stem cells (Martino et al. 2011; Bao and Song 2018). Nevertheless, it is becoming more and more evident that remarkable differences occur among animal species in regenerative capacities: in non-mammalian vertebrates (e.g., fish, amphibia, reptiles) stem cells are quite abundant and widespread in large portions of the CNS, thus granting continuous cell renewal, whereas in mammals the stem cell niches are highly restricted to only two-to-three small brain regions (Bonfanti 2011; Lindsey et al. 2018; Lange and Brand 2020; La Rosa and Bonfanti 2018; Vandestadt et al. 2021; Fig. 2). Accordingly, fish neurogenic processes can provide substantial possibilities for brain repair and regeneration after lesion (Lindsey et al. 2018; Lange and Brand 2020), whereas in mammals most regenerative capacity has been lost (Weil et al. 2008; Bonfanti 2011), the new neurons mainly playing a role in the postnatal maturation of specific neural circuits by sculpting their capability to learn from experience (Aimone et al. 2014; Semënov 2019; Kempermann 2019; Cushman et al. 2021; La Rosa and Bonfanti 2021; Fig. 2). Neurogenic plastic processes also differ among mammals, to serve the appropriate time course/functional adaptation of each species (Barker et al. 2011; Bonfanti and Charvet 2021) and follow diverse lifespans and related developmental schedules (Finlay and Darlington 1995; Workman et al. 2013).

In neurobiological research, the use of laboratory rodents as animal models has been prevalent, which has obscured our appreciation of the interspecies variation in different types of neurogenic plasticity. However, in recent years, these differences have started to come to light (Brenowitz and Zakon 2015; Faykoo-Martinez et al. 2017; La Rosa and Bonfanti 2018). Many researchers working exclusively on mice and rats make claims concerning the putative function(s) of adult neurogenesis by generalizing their conclusions to all mammals (see for example Gage 2019), yet many reports have revealed striking differences among species (Paredes et al. 2016; Parolisi et al. 2018; Sanai et al. 2011). Similarly, some scientists working on non-human primates (e.g., common marmosets, macaques), then generalize to “primates and humans” (see for example Hao et al. 2022). Yet, marmosets have relatively small (brain weight: 8,5 g) and lissencephalic brains (Gyrification index, GI: 1,18), which are remarkably different from other species of anthropoid primates with larger and more highly gyrencephalic brains (e.g., chimpanzee brain weight: 383 g, GI: 2,31; Zilles et al. 2013). In addition, compared to other anthropoid primates, marmosets exhibit unique life history traits. They have accelerated reproductive rates, shorter lifespans, earlier maturation, and regularly give birth to twins (Tardif et al. 2011; Preuss 2019).

**Fig. 3:**
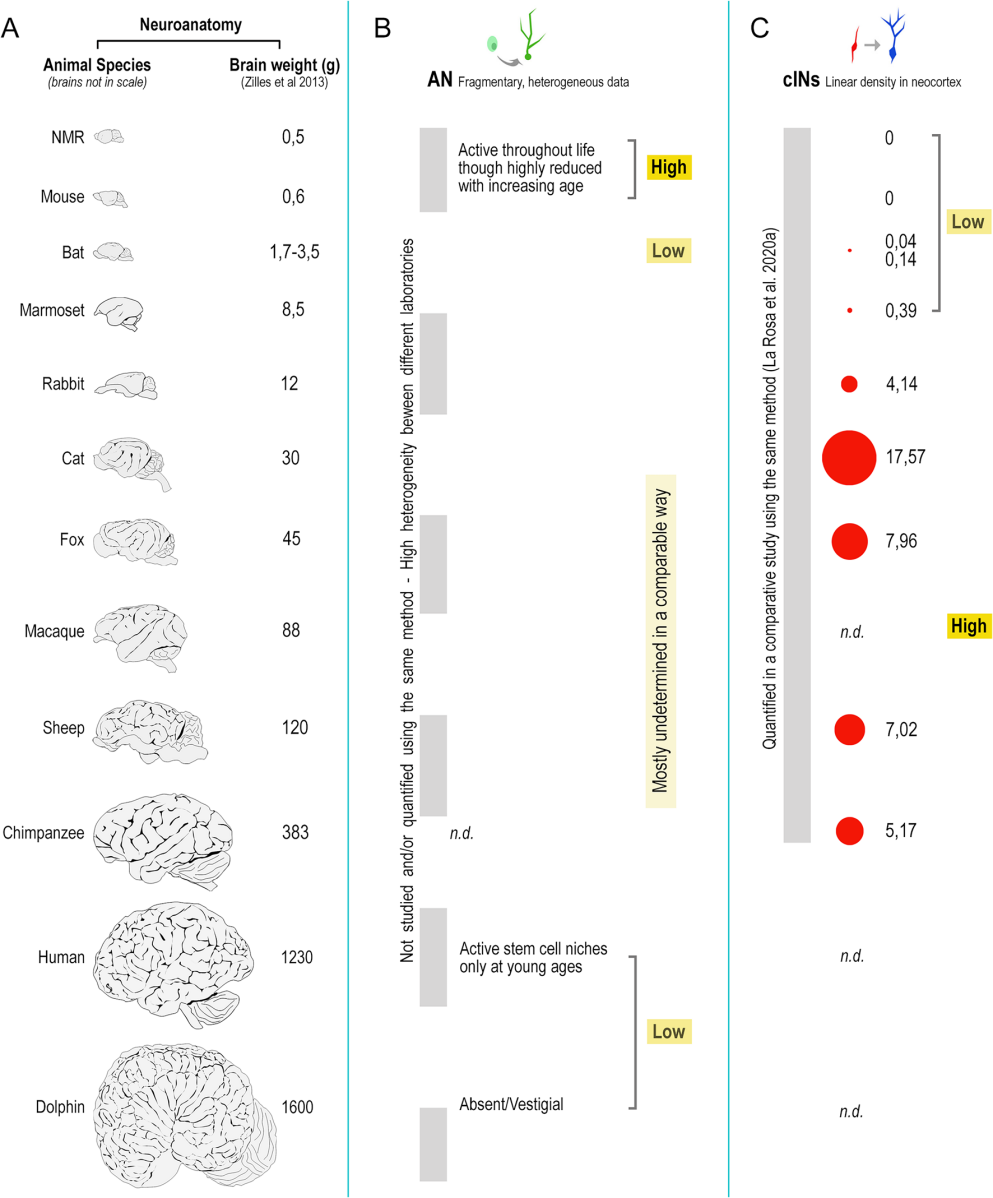
Phylogenetic variation of adult neurogenesis (AN) and cortical immature neurons (cINs) in mammals endowed with largely different neuroanatomy. Due to multiple difficulties in conducting large scale comparative studies, data are still fragmentary. **A**, brains of different mammals are represented from smaller to larger (brain weight reported on the right; not in scale). **B**, for adult neurogenesis, despite the existence of comparative studies, a lack of comparable quantitative analyses extended to many species makes it difficult a real comparison of their rates (uneven grey line). Available data reveal remarkable differences at the extremes: high rates in laboratory rodents vs. low rates, or even vestigial presence, in large-brained species. The main aspects of heterogeneity (mostly qualitative) are reported in the AN column C, for cINs, despite the overall scarcity of studies, a comparative, quantitative analysis has been performed on 10 mammalian species by using the same method; red dots of different sizes graphically represent the different cIN amount in the neocortex of different mammals (numbers indicate the median of linear densities in cortical layer II, as reported in La Rosa et al. 2020a)

**Fig. 4:**
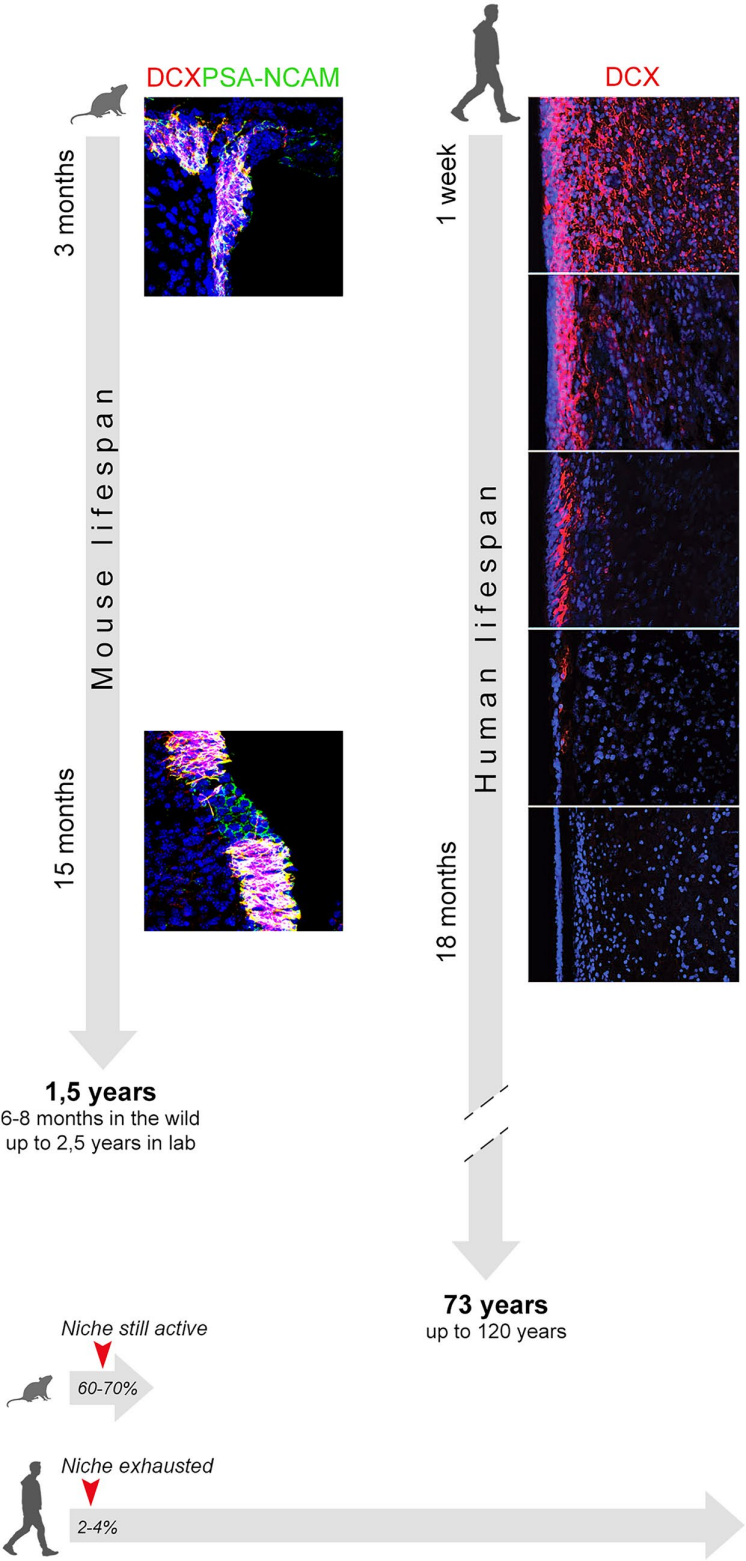
Remarkable variation in neurogenic activity in the forebrain subventricular zone stem cell niche of mice and humans. Substantial production of neuroblasts (identified by the expression of immature neuronal markers and cell proliferation—see also Fig. 1) is still present at 15 months, representing an advanced age in the mouse lifespan (around 50–70%). By contrast, the same neurogenic site dramatically drops its activity at very early stages (around 18 months), representing a very small percentage of the entire lifespan (around 2–3%). Images reproduced with permission from Ghibaudi et al. 2023a (mouse, left) and Sanai et al. 2011 (human, right)

The protracted generation of neurons during postnatal and adult stages has been identified as not merely a brain function, but rather as a “tool” that the brain can utilize to enhance specific functions. The specific functions that benefit from this neuronal generation can differ significantly among species and their socioecological adaptations (Barker et al. 2011). In one of the most elegant review articles written on this subject, Barker et al. (2011) state that “the function of adult neurogenesis is a task-dependent specialization”, so that comparative analysis in widely different species can help to understand neurogenesis as an evolutionarily conserved trait to meet ecological pressures. On this basis, we should “seek multiple explanations for the adaptive significance of adult neurogenesis and how particular ecological needs and evolutionary pathways have directed its function, where it occurs” (Barker et al. 2011). Indeed, remarkable differences do exist in the duration, location, type, and rate of plastic changes in different mammals (Lipp and Bonfanti 2016; Paredes et al. 2016; Palazzo et al. 2018). Recent comparative analyses carried out in rodents and non-rodent mammals have started to reveal possible phylogenetic trends for such variation, suggesting that different animal lineages display evolutionary specializations. Despite technical limits and some controversial data, results mostly converge to indicate that neurogenesis is almost entirely absent in regions of the adult human brain whereas neuronal addition continues into adult life in rodents (reviewed in Paredes et al. 2016; Parolisi et al. 2018; Sorrells et al. 2021; Duque and Spector 2019). The forebrain stem cell niche of the lateral ventricle subventricular zone (SVZ, providing new neurons for the olfactory bulb) is a striking example of interspecies difference (see below). On the other hand, a novel population of cortical “immature” neurons that are generated prenatally, then delaying their maturation and “awakening” during adulthood, are significantly more abundant in large-brained, non-rodent species (Palazzo et al. 2018; La Rosa et al. 2020a; Figs. 1, 2, 3, 4). The hypothesis that various forms of plasticity may arise due to evolutionary trade-offs linked to brain size and other neuroanatomical adaptations is gaining momentum. In this review article, we aim to provide a comprehensive overview and analysis of the existing data, while also placing them in a phylogenetic context. We will emphasize the gaps in our knowledge that continue to hinder a universally shared understanding of these topics. Understanding the implications of these trade-offs in different forms of neuronal plasticity has significant implications for mammals with varying brain sizes and adaptations. It can shed light on how different species have evolved distinct strategies to optimize cognitive abilities, sensory processing, and behavioral flexibility. By examining these trade-offs, we can gain valuable insights into the evolutionary mechanisms underlying the diversity of mammalian brains and their functional capacities.

## Heterogeneity of neurogenic processes: newly generated and non-newly generated “immature” cells coexist in adult brains

Before addressing phylogenetic variation of neurogenic processes, we summarize recent developments in the field that are changing our view about the possibility for adult brains to add new neurons through life. Until recently, efforts in developmental neurobiology have been mostly focused on stem cell-driven neurogenesis (Bonfanti and Peretto, 2011; Aimone et al. 2014; Bond et al. 2015; Lim and Alvarez-Buylla 2016; Kempermann 2019; Obernier and Alvarez-Buylla 2019). The discovery of adult mammalian neurogenesis raised new hopes to develop therapeutic strategies for neurological disorders (Martino et al. 2011; Bao and Song 2018). A huge number of reports have been published in the last 30 years (> 13,000 papers in PubMed) increasing our knowledge of the genesis, differentiation, integration, and modulation of new neurons in specific “neurogenic sites” located in restricted brain regions (mostly the olfactory bulb and hippocampus; Aimone et al. 2014; Lim and Alvarez-Buylla 2016). Over the years, other regions were proposed to host “non canonical” neurogenic processes, a finding which became more evident when different mammalian (non-rodent) species were analyzed (Ponti et al. 2006, 2008, 2010; Luzzati et al. 2006; Barker et al. 2011; Feliciano et al. 2015; Amrein 2015). In parallel, it was suggested that even in “canonical” neurogenic sites, remarkable variation can exist depending on the animal species and/or ages considered (Sanai et al. 2011; Patzke et al. 2015; Lipp and Bonfanti 2016; Parolisi et al. 2017, 2018; Cipriani et al. 2018; Sorrells et al. 2018; see dedicated section below). All these variables increased the complexity of the field, sometimes creating confusion in the interpretation of results (Lipp and Bonfanti 2016; Oppenheim 2019; Duque et al. 2022). Apart from technical considerations regarding common pitfalls in the reliable detection of cell genesis, which have been addressed elsewhere (Duque and Spector 2019), some bona fide mistakes can be generated by erroneous interpretation of markers of immaturity that are commonly used in the study of adult neurogenesis, such as the cytoskeletal protein doublecortin (DCX; Nacher et al. 2001) and the polysialylated, low-adhesive form of the Neural Cell Adhesion Molecule (PSA-NCAM; Acheson et al. 1991). Since these proteins are transiently expressed by neuroblasts produced in the neurogenic niches, they were universally considered as reliable markers, or proxies, for neurogenesis and “an alternative to bromodeoxyuridine (BrdU) labeling” (Brown et al. 2003; Bonfanti 2006). On this basis, neurogenesis was reported to occur in various brain regions out of the canonical stem cell niches, some of which were found to host DCX-immunoreactive (DCX^+^) cells that are not associated with cell division and neurogenesis (Bonfanti and Nacher 2012; Nacher and Bonfanti 2015; König et al. 2016; reviewed in La Rosa et al. 2020b). In recent years, researchers have discovered a possible explanation for these observations in layer II of the piriform cortex (paleocortex). A population of cortical “immature” neurons (cINs) have been identified that are not newly generated but are instead born prenatally and continue to exhibit markers of immaturity throughout adulthood (Gómez-Climent et al. 2008; Klempin et al. 2011; Bonfanti and Nacher 2012; König et al. 2016; Bonfanti and Seki 2021; Fig. 1C). These cells undergo delayed maturation and might represent a new form of “neurogenesis without division”, involving “dormant” neural elements “frozen in a stand-by mode” and sharing the same markers of immaturity with newly born neurons (Gómez-Climent et al. 2008; Bonfanti and Nacher 2012; König et al. 2016; Piumatti et al. 2018; Rotheneichner et al. 2018; La Rosa et al. 2020b). Using DCX-Cre-ERT2/Flox-EGFP transgenic mice, in which the green fluorescent protein (GFP) is permanently expressed in DCX^+^ cells and in their progeny following tamoxifen administration, it was confirmed that most cINs mature throughout life into glutamatergic neurons (Rotheneichner et al. 2018), and can be integrated into the pre-existing piriform cortex network (Benedetti et al. 2020; Fig. 1). Although the role, fate, and significance of this neuronal population, as well as the mechanism leading to block their maturation (and to wake up them later) are still unknown, the cINs can be considered as highly plastic cells which might represent a reservoir of young neurons in adult brains (Rotheneichner et al. 2018; La Rosa et al. 2019, 2020a, b; Benedetti and Couillard-Despres 2022).

Similarly, DCX^+^ “immature” cells are detectable in subcortical regions (amygdala, claustrum, white matter), also in this case having originally been identified as either neurogenic events (Bernier et al. 2002; Marlatt et al. 2011; Jhaveri et al. 2018) or “immature” neurons (Fudge 2004; Martí-Mengual et al. 2013; Sorrells et al. 2019; Chareyron et al. 2021; reviewed in Ghibaudi and Bonfanti 2022). At these locations our current understanding is incomplete, and further studies are needed to correctly classify these DCX^+^ cells (Ghibaudi and Bonfanti 2022; see below). For this reason, the present review will mainly focus on the cINs.

The complex issue of the different types of young neurons has slowly emerged across the years within the well-established field of adult neurogenesis (Bonfanti and Seki 2021), and it is rapidly evolving (Benedetti and Couillard-Despres 2022; Ghibaudi and Bonfanti 2022). It is now clear that beside the newly born cell populations produced in the stem cell niches, many DCX^+^ neurons in adult brains appear to be in a state of protracted or arrested maturation, maintaining immature marker expression and a simple morphology for long time. In mammalian brains, these cell populations coexist with adult neurogenic processes, yet their relative occurrence, distribution and amount can vary remarkably across different species (La Rosa et al. 2020a; Ghibaudi and Bonfanti 2022). While comprehensive studies and comparable data on such phylogenetic variation are scarce and largely incomplete (Fig. 3), the following sections will provide a summary of our current understanding of this subject and the potential evolutionary trade-offs that take place in mammal brain evolution, involving different neurogenic strategies.

## Phylogenetic variation in canonical adult neurogenesis

### Heterogeneity of approaches can make it difficult to compare adult neurogenesis across species

The issue of occurrence, location and rate of canonical adult neurogenesis in different species is far from being solved, due to the lack of quantitative data obtained in a systematic, comparable way (Fig. 3B). Only a small number out of 700,000 articles published in the neuroscience field since the year 2000, and more than 13,000 articles published on adult neurogenesis since the 1990s, used a comparative approach, the vast majority of the investigations having been performed on laboratory rodents (Lipp and Bonfanti 2016; Cozzi et al. 2020). Some comparative studies describing differences in adult neurogenesis in different mammalian groups are available (see for example Barker et al. 2011; Patzke et al. 2015; Amrein 2015; Paredes et al. 2016; Parolisi et al. 2018), nevertheless the original reports differ in various significant ways, including age of subjects, brain regions examined, source of material, type and time of tissue fixation, postmortem intervals, type of markers, antibodies and detection method employed, type of quantitative analyses, and aim of the study (Zhao and van Praag 2020; Ghibaudi et al. 2023a). Though some researchers point specifically to tissue fixation and postmortem interval as a source of variation in observations (Moreno-Jiménez et al. 2021), in a recent study we highlighted that other variables are important when dealing with comparative immunocytochemical detection of plasticity-related markers (Ghibaudi et al. 2023a). These variables are mainly represented by the choice and availability of primary antibodies (that can react very differently in different species), and by the existence of actual interspecies differences in the presence and distribution of antigens. In our extended study, involving six widely different mammalian species, spanning from mice to humans, we showed that very similar results can be obtained in tissues treated with different types of fixation (including intracardiac perfusion and tissue immersion) and with different postmortem intervals, while both absence of staining or non-specific staining can occur when using different commercially available antibodies (Ghibaudi et al. 2023a). Moreover, in our experience on DCX^+^ cortical immature neuron detection in animal species endowed with widely different brain sizes, the highest numbers of these cells were found in the largest brains, namely in those tissues that are technically more difficult to be fixed and processed (Piumatti et al. 2018; La Rosa et al. 2020a). Finally, by using an in situ hybridization with RNA probe (RNAscope), absence of staining or non-specific staining for DCX was found to be frequent in human brain tissues treated with several antibodies, most of them being raised to work in mice (Ghibaudi et al. 2023a).

Age is another variable that can affect the rate of neurogenesis (Ben Abdallah et al. 2010; Semënov 2021), and animal species widely differ in their length of development and lifespan (Snyder 2019; Bonfanti and Charvet 2021). In other words, the species matters more than often acknowledged.

For obvious reasons linked to the scarce availability of well-fixed tissues for large-brained mammals, to the technical difficulties encountered in their analysis and related ethical issues, comparative studies encompassing multiple species are rare or limited to different rodents and mouse strains (van Dijk et al. 2019). Also, accurate longitudinal studies on the rate of cell division in the neurogenic sites at different ages are mostly limited to single species (mostly rodents; Ben Abdallah et al. 2010; Semënov 2021). This is due to the lack of reliable tools to trace the history and fate of the newly generated elements in vivo, through time, in animal species that are protected by ethical guidelines such as elephants, whales, great apes, and others. In addition, a common bias that has come to light in recent years consists of neuronal populations sharing the same markers of immaturity, e.g., DCX and PSA-NCAM: the newly generated neurons (produced in the process of stem cell-driven adult neurogenesis), and the “immature” neurons frozen in a state of arrested maturation (but having lost the capacity to undergo cell division; see La Rosa et al. 2020b, and below), the abovementioned markers being previously considered as specific markers for adult neurogenesis (Brown et al. 2003; see above). As an example, the detection of DCX^+^ neurons in the adult human dentate gyrus has been interpreted as adult neurogenesis (Moreno-Jiménez et al. 2019), even in the absence of substantial cell division (Sorrells et al. 2021), thus being rather ascribable to persistent immature neurons (Zhou et al. 2022).

Finally, considering that a substantial decrease in the genesis of new neurons does occur in all species with increasing ages, it is not always easy to establish a comparison between widely different mammals, due to their different neurodevelopmental schedules and maturational states (Workman et al. 2013; Bonfanti and Charvet 2021).

In summary, we are still far from reaching a complete and reliable, comparative mapping of adult neurogenesis occurrence, distribution and rate in widely different mammals (especially concerning the rate of cell division giving rise to the new neurons, with respect to the immaturity marker detection), although a general trend implying evolutionary trade-offs is starting to emerge.

### Despite heterogeneity, a general trend of reduction in adult neurogenesis from small-brained to large-brained species is emerging

Although the comparative data on adult neurogenesis in mammals is incomplete and varied, the available evidence strongly suggests that this process may have undergone significant evolutionary changes across different phylogenetic groups. Our current knowledge regarding such variation is mainly qualitative, being based on: (i) observations provided by histological and immunocytochemical studies concerning the existence of morphological and molecular features typical of stem cell niches in the neurogenic sites (Sanai et al. 2011; Sorrells et al. 2018, 2021; Fig. 3), and (ii) a small number of quantitative studies of the rates of cell division (Fig. 3), with all the limits described above. An extreme example is represented by the dramatic drop in neurogenic activity within the olfactory system of some large-brained mammals (Fig. 4). Dolphins, which are large-brained, long-living aquatic mammals lacking a sense of smell, exhibit a vestigial and largely inactive subventricular zone (SVZ) at birth (Parolisi et al. 2017, 2018). This observation was obtained after careful analysis of 10 postmortem dolphin brains (5 neonates and 5 adults), by using internal positive controls for DCX and Ki-67 antigen in the highly proliferating external granule layer of the cerebellum (Parolisi et al. 2015, 2017). In humans, the SVZ substantially ceases to produce newly born neuroblasts for the olfactory bulb around two years of age (Sanai et al. 2011), which represents a relatively early stage in the human lifespan (Fig. 4).

The current controversy concerning the occurrence/rate of adult hippocampal neurogenesis in humans (Moreno-Jiménez et al. 2021; Sorrells et al. 2021), is raised by the contrast between the finding of DCX^+^ and PSA-NCAM^+^ neurons in the adult hippocampus (Mikkonen et al. 1998; Boldrini et al. 2018; Moreno-Jimenez et al., 2019; Tobin et al. 2019; Seki et al. 2019, 2020), in the absence of a morphologically-recognizable stem cell niche (Sorrells et al. 2018) and with very low levels of cell division (reported by most studies, though with different methods; see for example Sorrells et al. 2018; Cipriani et al. 2018; Moreno-Jimenez et al., 2019; Seki et al. 2019). This discrepancy has at present no clear explanation, yet, might be partially understood as a general trend for a higher occurrence of “immature” neurons in large-brained mammals (Palazzo et al. 2018; discussed in the next paragraph) and/or by possible processes of “dematuration” because of inflammation or pathological states in older adult individuals (Hagihara et al. 2019).

One of the recognized causes for age-related reduction is surely stem cell depletion, consisting of a mix of real, progressive exhaustion of the stem cell pool (reduction of the stem cell number; Encinas et al. 2011; Obernier et al., 2018) and entry in stem cell quiescence (Urbán et al. 2019). It has been proposed that similar mechanisms may limit neurogenesis to infancy in animals with very long lifespans, like humans (Obernier et al., 2018). In both neurogenic sites, with some differences in the slope of reduction between SVZ and hippocampus, a substantial genesis of new neurons is a juvenile event (Ben Abdallah et al. 2010; Semënov 2021), being influenced by lifespan extension and its impact on the timing of neurodevelopmental events across species (Snyder 2019; Charvet and Finlay 2018).

## Phylogenetic variation in “immature” neurons

**Fig. 5:**
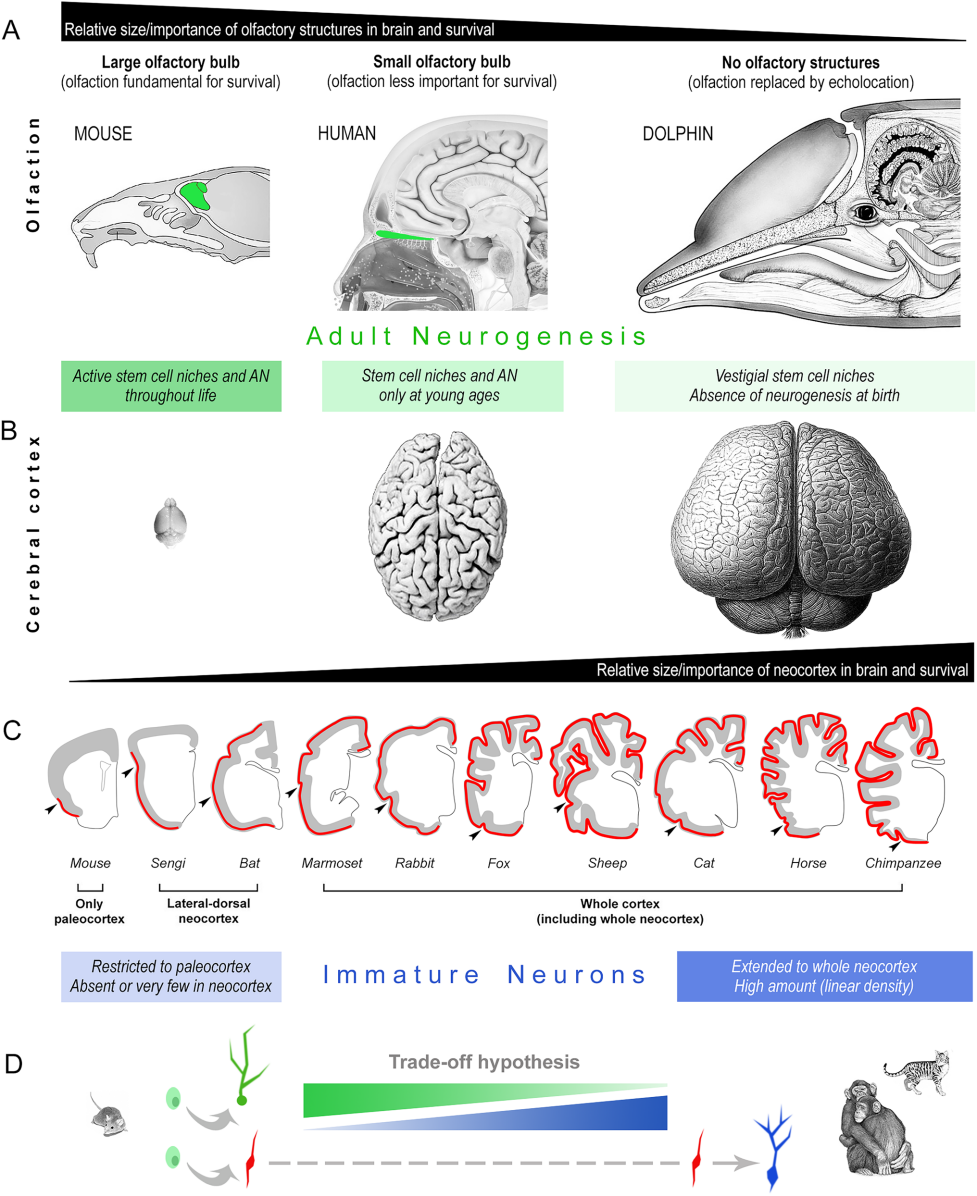
Anatomical and functional aspects at the basis of the evolutionary trade-off hypothesis to explain the relative occurrence between different types of brain structural plasticity (adult neurogenesis and cortical immature neurons) in mammals. **A**, **B**, different importance of certain brain regions/functions for navigation and survival: small-brained rodents rely mostly on olfaction, whereas large-brained, gyrencephalic species rely mostly on widely expanded cerebral cortex (neocortex). C, immature neurons are more widespread (in the cortical mantle) and abundant (in terms of cell density) in large-brained, gyrencephalic species with respect to rodents. They would have been favored by evolution to place a process of “neurogenesis without division” (i.e., the addition of new functional neurons) in brain regions not endowed with stem cell-driven neurogenesis (e.g., cerebral cortex). D, the prevalence of immature neurons in the cortex of large-brained species and of stem cell-driven neurogenesis in the neurogenic sites of rodents suggests a trade-off in different types of neurogenic plasticity

The topic of cortical immature neurons (cINs), along with the concept of “neurogenesis without division,” is relatively new, and thus, still not fully explored (Bonfanti and Seki 2021; Benedetti and Couillard-Despres, 2022). Many questions remain unanswered, among which are the molecular and cellular mechanisms that allow these neurons to halt their maturation before birth and subsequently “awaken” during adulthood. Also, their prevalence throughout the brain is not yet precisely known (Ghibaudi and Bonfanti 2022; Page et al. 2022), and it remains unclear whether they can be activated in response to injury, inflammation, or neurological disorders (excluding recent reports on subcortical, putative immature cell populations in macaques; Chareyron et al. 2021, discussed below). In parallel, some insight has been gained about the phylogenetic variation of cINs through systematic investigation of the cortex of different mammalian species widely varying in brain size, gyrencephaly and socioecological features, providing an unexpected twist in our understanding of comparative neuroplasticity (Piumatti et al. 2018; La Rosa et al. 2020a; Fig. 3C). Previous reports indicated that in laboratory rodents cINs are highly restricted to the piriform and entorhinal regions of the paleocortex (Seki and Arai 1991; Bonfanti et al. 1992; Nacher et al. 2001), though DCX^+^ neurons were also observed in the neocortex of some mammals, including guinea pigs, rabbits, and cats (Cai et al. 2009; Zhang et al. 2009; Varea et al. 2011; Luzzati et al. 2009; Xiong et al. 2008). In experiments using pregnant sheep treated with BrdU injections, with subsequent analysis of the lamb’s brains, we showed that most of the DCX^+^ neurons in the cerebral cortex (including neocortex), as well as some in subcortical regions (amygdala and claustrum), were generated prenatally, while still expressing markers for immaturity (Piumatti et al. 2018). Hence differences exist in the anatomical distribution of cINs among mammals, suggesting a more widespread presence in large-brained gyrencephalic species (Palazzo et al. 2018). Since most comparative studies on this subject were carried out on single animal species, by different laboratories, and using different methods of tissue processing and cell counting, we decided to perform a comparative study across mammals by addressing the occurrence, distribution, and amount of cINs in the whole cortical mantle (La Rosa et al. 2020a). In that study, 84 brains were processed by using the same method to identify and count the cortical layer II DCX^+^ cINs to obtain a linear density (number of cINs/mm of cortical layer II; Fig. 3C). The analysis revealed an extension of the presence of cINs from paleocortex in rodents to the entire neocortical mantle in gyrencephalic mammals (Fig. 5), with remarkable variation in cell density (one order of magnitude when comparing the group of small-brained species with large-brained ones; La Rosa et al. 2020a; Figs. 3, 4, 5). The presence of DCX^+^ cINs has been confirmed in the cerebral cortex of humans (Knoth et al. 2010; Liu et al. 2018; Sorrells et al. 2021; Coviello et al. 2022; Ghibaudi et al. 2023a; Li et al. 2023). Though systematic quantitative data in humans are not yet available (comparable cell density), it has been shown that these neurons cover layer II of the entire cortical mantle, being preserved at adult and old ages (Li et al. 2023). Thus, it appears that the cINs could grant a reservoir of young cells for the neocortex of large-brained species. For the highly complex cerebral cortices of these mammals, to rely on pre-existing neurons that can be added functionally throughout life might be an evolutionarily advantageous, energetically inexpensive solution for overcoming the lack of stem cells and progenitor cells (La Rosa and Bonfanti 2021). This aspect might also be linked to increased lifespans, since most large-brained mammals are also long-living with respect to mice. Interestingly, a prolonged maturation of the newlyborn neurons (up to 3–5 months) has been found in the naked mole rat, a rodent reaching thirty years of age and showing maturational features of large-brained, long-living mammals (Faykoo-Martinez et al. 2022).

Several reports indicate that “immature” neurons might exist in subcortical regions as well, also with significant interspecies variation (Chareyron et al. 2021; Ghibaudi and Bonfanti 2022; Page et al. 2022). For example, in a detailed study conducted on the human amygdala from embryogenesis to adulthood (Sorrells et al. 2019), an immature neuronal cell population (DCX^+^, PSA-NCAM^+^) that maintains a small size and a simple morphology for decades was found in the basolateral nucleus. The authors suggest that a portion of these cells undergo maturation as excitatory neurons (TBR1^+^/VGLUT2^+^), mainly during adolescence. Yet, some DCX^+^/PSANCAM^+^ cells persist even at older ages, in association with Ki67^+^ nuclei. These proliferating cells did not overlap with DCX^+^/PSANCAM^+^ cells, being primarily associated with blood vessels or oligodendrocytes (Sorrells et al. 2019). In contrast to the persistent presence of immature cells, the authors showed a sharp decline in the Ki67^+^ cells population in the first years of life.

The same research group recently studied the DCX^+^ cells of the amygdala in mice (only a few cells are detectable in the rodents studied to date), revealing that they can migrate to the piriform cortex at early postnatal stages, to add as glutamatergic excitatory neurons to the cINs already in place (Alderman et al. 2022). By using embryonic BrdU birth dating, this study confirms that immature neurons in the amygdala are generated prenatally, with results similar to those obtained by Gómez-Climent et al. (2008) for cINs, thus extending the concept of INs to subcortical regions. Though we still lack systematic comparative analyses, the data obtained in humans and mice indicate that subcortical immature neurons may also display interspecies variation.

Changes in the immature neuronal population of the amygdala have been described after bilateral hippocampal lesion in neonatal and adult monkeys (*Macaca mulatta*; Chareyron et al. 2016). The lesion-induced increase in the number of mature neurons in the amygdala has been interpreted as the product of different processes, including the maturation of resident immature cells, a migration of immature neurons from the paralaminar nucleus to other nuclei, or from a stream of neuroblasts originating in the SVZ (Bernier et al. 2002; Chareyron et al. 2016).

Overall, the amygdala of primates continues to undergo structural changes during essential formative years in the juvenile period and later in life, both in physiological and pathological conditions, mostly through maintenance of populations of immature excitatory neurons.

## Hypotheses on a possible evolutionary trade-off between different types of neurogenic plasticity

Based on the evidence of phylogenetic variation in stem cell-driven neurogenesis and non-dividing “immature” neurons, it is likely that evolutionary pressures associated with ecological niche or neurodevelopmental constraints have led to the selection of different types of plasticity in various species and brain regions. This suggests a “trade-off”, which refers to a situation where compromise occurs between two or more traits that offer distinct benefits but cannot be fully optimized concurrently. Such compromises can arise due to limited resources or energy that must be allocated among competing demands, or due to anatomical or developmental limitations (Heldstab et al. 2022).

The concept of a trade-off implies that the balance of resource allocation can shift between different options without necessarily indicating an exclusive commitment to one over the other (Noordwijdk and Jong, 1986). Consequently, evolutionary processes might favor specific forms of plasticity in certain species or brain regions, depending on the ecological pressures and functional demands they face. We hypothesize that trade-offs play a crucial role in shaping the evolutionary trajectory of neural plasticity and contribute to the remarkable diversity observed across species. Here we propose that trade-offs can be observed in forms of neurogenic processes across mammal species between those that require or do not the presence of stem/progenitor cell division.

### Brain size and balance in the allocation of resources

It is important to consider the factors that influence the occurrence of trade-offs in evolutionary biology. Limited resources, such as energy, nutrients, or developmental timing can pose constraints on an organism's ability to optimize multiple traits simultaneously. These constraints lead to the need to balance the allocation of resources so that one trait may come at the expense of another. For example, in the context of neurogenesis, maintaining a larger pool of stem cells for continuous regeneration may come at the cost of other energy-demanding processes, such as enhancing synaptic plasticity or cognitive functions (Walton et al. 2012).

Additionally, anatomical constraints can also contribute to trade-offs. The physical structure and organization of an organism's brain can impose limitations on the optimization of multiple traits. For instance, brain regions with limited space or specialized functions may prioritize specific forms of plasticity that are most beneficial for their ecological niche, while compromising on others (Charvet and Finlay 2018).

Nevertheless, it is important to note that evolution, as a process, is not linear, progressive, or predictable. While it operates through natural selection and the accumulation of advantageous traits over time, it also encompasses elements of randomness through neutral drift and contingent exceptions that defy straightforward explanations. The interplay of genetic variation, environmental factors, and chance events introduces a level of unpredictability (e.g., bats are small-brained mammals with reduced adult neurogenesis, or naked mole rats which are long-living rodents with abundant adult neurogenesis; Amrein et al. 2007; Penz et al. 2015). However, amid this complexity, certain trends and patterns can be identified. These trends are governed by the balance of energetic allocation and developmental constraints, ultimately shaping variation in brain structure across species and the capacity for different forms of plasticity.

In the case of neurogenic plasticity, the following variables are relevant: (i) the type of plasticity (e.g., stem cell-driven neurogenesis and non-dividing immature neurons); (ii) the anatomical region hosting plasticity that is linked to specific functions (e.g., canonical neurogenic site linked to olfaction and cerebral cortex linked to high-order computational capabilities); (iii) the phylogenetic lineage of the species and their brain size.

Adult neurogenesis in large mammal brains is subject to various energetic costs and developmental constraints. The biosynthetic process of generating new neurons requires substantial metabolic resources, including glucose and oxygen, which can impose a significant burden on the energy budget of the brain (Bauernfeind and Babbitt 2020). Large mammal brains may face challenges in allocating sufficient resources for neurogenesis while maintaining other essential functions. Additionally, the developmental constraints associated with large brain size can limit the spatial and temporal availability of neurogenic niches, where new neurons are generated (Patzke et al. 2015; Charvet and Finlay 2018; Martinez-Cerdeno et al., 2018; Duque and Spector 2019). This may restrict the extent and duration of adult neurogenesis in large mammals.

**Fig. 6:**
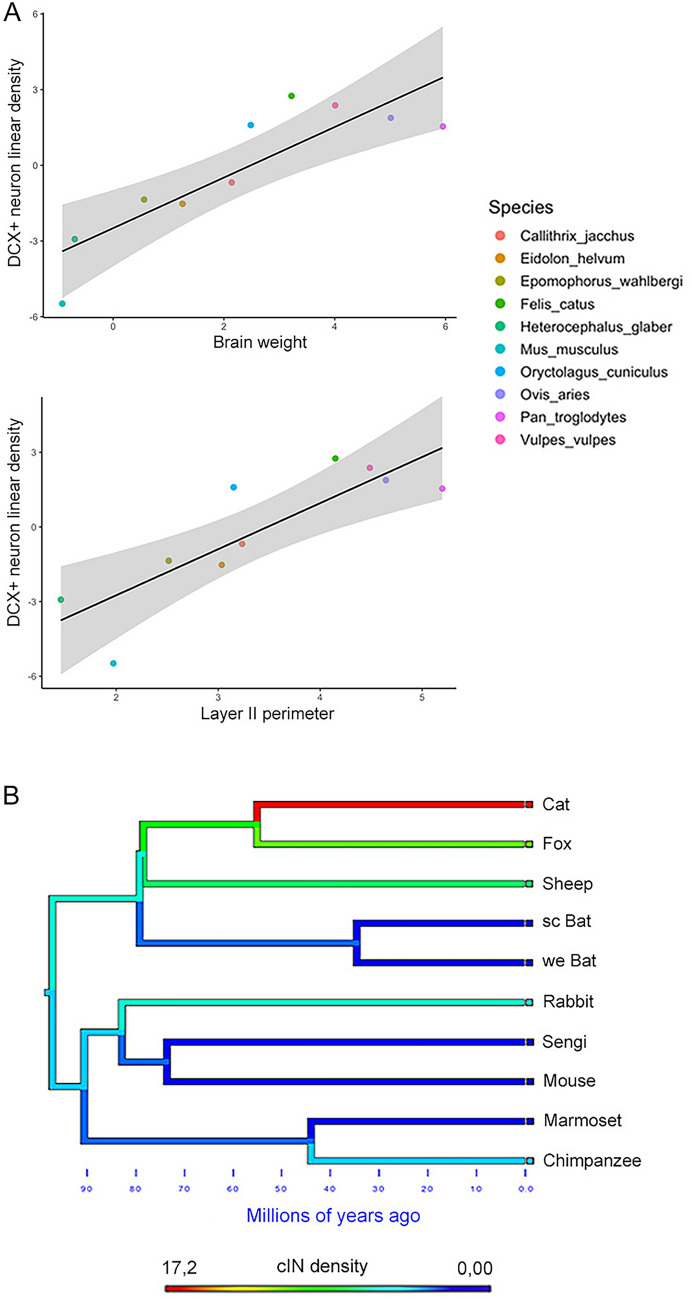
The amount of cortical immature neurons is linked to brain size. **A**, phylogenetic generalized least squares regression (PGLS) showing that linear density of neocortical DCX^+^ neurons covaries significantly with brain weight and cortical layer II perimeter. **B**, map of character evolution on the phylogenetic tree illustrating the independent emergence of neocortical DCX^+^ neuron densities in the mammalian species considered. Reproduced with permission from eLife, Elife Sciences Publications (La Rosa et al. 2020a)

By contrast, the prenatally generated, non-dividing cortical “immature” neurons, which do not require stem/progenitor cells to occur as undifferentiated elements within the mature cortex, are far more abundant and widespread in large-brained mammals (La Rosa et al. 2020a; Fig. 6), likely representing a “low energy cost”, alternative form of neurogenic plasticity. For instance, it has been shown that virtually all the cINs eventually awaken and pursue their fate to functional integration across the animal lifespan (Benedetti et al., 2023). On the other hand, it is well known that at least 60% of the cells produced in canonical adult neurogenesis will die by apoptosis during the first week after division, and others will be selectively lost while trying to reach their target (Sierra et al. 2010; Pilz et al. 2018), so that only a few cells will eventually integrate. In addition, the cINs are already in place within their destination (layer II) since the last phases of embryogenesis, hence not needing migration, and are ensheated by astrocytic lamellae, having only a few or no synapses (Gomez-Climent et al., 2008). Of course, it is far from clear how these cells can survive apparently isolated from the surrounding neuropil, yet these features might represent an advantageous source for providing new neurons to locations across the entire cortical surface, in the absence of active stem cell niches.

### From olfaction to neocortex: the hypothesis of navigation adapted to plasticity

Recent theories propose that the origin of the neocortex in early mammals resulted from behavioral adaptations related to olfaction-mediated goal-directed and navigational behaviors, accompanied by integrated sensory map development, which in turn resulted in developmental changes in the distribution of cells and the formation of circuits in the telencephalon (Aboitiz et al. 2003; Aboitiz and Montiel 2015). Early mammals likely adopted a nocturnal and burrowing lifestyle, utilizing internal cues such as proprioceptive information in conjunction with sensory inputs from the olfactory and somatosensory systems for spatial navigation. In such conditions, orientation was predominantly based on one-dimensional maps that encoded sequences of events in a time series. These early mammals are thought to have relied heavily on their sense of smell, leading to an expansion of the olfactory bulb and olfactory cortex as brain size increased. Accordingly, selective pressures led to the emergence of an interface between olfactory-hippocampal networks, integrating somatosensory information for navigation (Kaas 2019).

As mammals diversified and occupied new ecological niches, including diurnal environments for some species, vision and audition provided additional information regarding distance and location. These senses are vital for generating accurate two-dimensional and time-independent spatial maps, providing more detailed information relevant to navigation (Buzsáki 2005; Eichenbaum 2014). Over time, the expanding neocortex played an increasingly prominent role in the formation of multimodal association networks and map-like representations of space (Aboitiz and Montiel 2015).

The current diversity of brain structure in mammals is extraordinary. Large-brained mammals with gyrencephalic brains often exhibit reduced olfactory bulb size or complete absence of olfactory bulbs, as observed in dolphins (Fig. 5; references in Parolisi et al. 2018). In contrast, smaller-brained mammals, including most rodents, possess prominent olfactory structures and relatively smooth neocortices. In these species, the activity of the periventricular neurogenic niche (SVZ) is impressive, providing thousands of new neurons/day for the olfactory bulb through the animal lifespan, and allowing experience-induced plasticity linked to olfaction (Lim and Alvarez-Buylla 2016; Lledo and Valley 2016; Fig. 4). By contrast, the SVZ neurogenic niche of humans is exhausted at very early postnatal stages, then leaving only a vestigial remnant (Sanai et al. 2011; Fig. 4). This anatomical variation reflects functional adaptations, with rodents heavily relying on olfaction, while larger mammals exploit the computational capabilities of their expanded neocortical circuits. The neocortex, characterized by six layers, undergoes remarkable elaboration in large-brained mammals with greater differentiation of specialized cortical fields that are important for sensorimotor integration and cognitive functions (Englund and Krubitzer 2022). Large-brained species with highly folded neocortices, such as primates, exhibit reduced dependence on olfaction, with their behavioral complexity predominantly linked to an extensively expanded neocortical mantle. This difference in reliance on olfaction and neocortical development could explain the lower levels of neurogenesis observed in these species, which primarily occur during the postnatal and juvenile stages to shape neural circuits through experiential learning (Semënov 2019; Kempermann 2019; Cushman et al. 2021). Consequently, a possible adaptation in large-brained, long-living mammals is the selection of non-dividing immature neurons (cINs) as a mechanism to provide a form of neurogenic plasticity in layer II of the cerebral cortex (as discussed in La Rosa et al. 2020a). This alternative mechanism for structural plasticity in highly expanded neocortices may serve an especially important functional role for species that lack abundance of active stem cells in the neurogenic sites, and may contribute to maintain neotenic features in brains with extended lifespans.

In considering the evolutionary reasons for the prevalence of diverse neurogenic processes, numerous questions arise regarding the role, mechanisms, and connectivity associated with plasticity linked to cINs. First, the observation that small-brained mammals exhibit this characteristic primarily in specific limbic areas, while other regions such as the sensory, motor, and association cortex lack it, suggests that a reservoir of cINs might not be a necessary supplement to synaptic plasticity in neocortical regions of these species. On the other hand, the existence of dormant neurons might have a role in large brains with expanded neocortices since the number of these cells is considerably greater than in small-brained rodents (see estimations below). Yet, the question arises: does the incorporation of a small number of new neurons over several years of an organism’s lifespan yield a significant functional benefit? The answer may involve a trade-off, as the number of dormant cINs significantly increases in larger brains with expanded neocortices, such as those in chimpanzees compared to rodents. For instance, the total number of cINs has been estimated at 36,000 (18,000 per hemisphere) in three-month-old mice (Ghibaudi et al. 2023b), whereas it reaches approximately 5 million (2.5 million per hemisphere) in chimpanzees (La Rosa et al. 2020a, b), representing a two-order-of-magnitude difference. Although the total number of cortical neurons has been estimated to be 5–7 million/hemisphere in mice (Herculano-Houzel et al. 2006), and 3, 7 billion/hemisphere in chimpanzees (Collins et al. 2016), namely a difference of three orders of magnitude, the difference in cIN density in the neocortex of mouse and chimpanzee is even more striking, with a five orders of magnitude increase in chimpanzees (La Rosa et al. 2020a). Despite an evident interspecies difference, whether adding a few cells for every thousands of existing neurons during the course of a lifetime can make a functional difference remains far from clear.

A third unresolved question pertains which connections these neurons might establish. It's worth noting that these immature neurons are found in layer II, a region considered to have a role in furnishing corticocortical association projections (and association with respect to other cortical layers), which may make it an ideal location for a neurogenesis-like process within the structurally stable cerebral cortex (La Rosa et al. 2020a). How these neurons orchestrate long-range axonal growth to reach their targets is a topic that requires further study.

Lastly, our current understanding of subcortical immature neurons strongly suggests that their numbers only moderately decline with age in gyrencephalic large-brained species, maintaining a substantial pool of immature cells (like a sort of neoteny) even in advanced life stages. This phenomenon has been observed in sheep (Piumatti et al. 2018) and humans (Sorrells et al. 2019) and has been discussed in Ghibaudi et al. (2023b). This observation raises intriguing hypotheses about the functional role of immature neurons, which may not solely rely on their structural integration. It’s possible that not all these neurons become active; some may remain immature and exert paracrine effects, such as trophic, neuroprotective, or bystander influences, on mature neural networks.

The presence of a trade-off between stem cell-driven neurogenesis and immature, dormant cINs in favor of the latter in gyrencephalic, long-lived mammals calls for further fundamental and comparative research. This research should aim to comprehensively elucidate all aspects of this fascinating mechanism in brain plasticity, holding significant translational implications.

## Conclusions

Biomedical research, including the neurosciences, is largely conducted on laboratory animal models, mostly mice and rats (Brenowitz and Zakon 2015; Bolker 2012, 2017; Faykoo-Martinez et al. 2017; La Rosa and Bonfanti 2018; Cozzi et al. 2020). Comparative studies using different mammalian species represent a small fraction of current research, although interest in the neurobiology of non-rodent mammals, including large-brained species and humans, has been increasing. One of the reasons surely is due to recent findings highlighting remarkable interspecies differences in the occurrence, extension, and rate of neural plastic processes (this review article). Comparative studies can help us to better understand the possible trade-offs that occur during evolution between different types of plasticity, thus providing a more comprehensive picture of these processes in mammals to avoid confusion and misinterpretation coming from the exclusive use of rodents as animal models (Lipp and Bonfanti 2016; Faykoo-Martinez et al. 2017). Do these differences have consequences for cognition, learning, capacity to recover from injury, or some other function? According to Jessica Bolker (2012) “disparities between mice and humans may help to explain why the millions of dollars spent on basic research have yielded frustratingly few clinical advances”. Now we know that disparities between reparative and physiological (homeostatic) plasticity, as well as between adult neurogenesis and “immature” (dormant) neurons, may contribute to explain these difficulties in translation.

Whatever the evolutionary reason, the differences in brain structural plasticity among animal species do exist, are remarkable, and indicate a gain in widespread adaptive plasticity at the expenses of loss in reparative and regenerative capability. This diversity may potentially frustrate therapeutic translation from animal models, but there is reason to be optimistic that the comparative perspective will bring exciting breakthroughs in our understanding of the role of plasticity in driving postnatal brain development and maintaining a healthy and efficient brain throughout life.

## Data Availability

Enquiries about data availability should be directed to the authors.
